# The future of Cardiothoracic surgery in Artificial intelligence

**DOI:** 10.1016/j.amsu.2022.104251

**Published:** 2022-07-31

**Authors:** Hassan Mumtaz, Muhammad Saqib, Farrukh Ansar, Durafshan Zargar, Madiha Hameed, Mohammad Hasan, Pakiza Muskan

**Affiliations:** a Maroof International Hospital, Islamabad Public Health Scholar: Health Services Academy, Islamabad, Pakistan; b Khyber Medical College, Peshawar Hospital, Khyber Teaching Hospital, Peshawar, Pakistan; c Northwest School of Medicine, Peshawar, Pakistan; d Ayub Medical College, Abbottabad, Pakistan; e Jinnah Postgraduate Medical Center (JPMC), Pakistan; f Fauji Foundation University, Islamabad, Pakistan

## Abstract

Humans' great and quick technological breakthroughs in the previous decade have undoubtedly influenced how surgical procedures are executed in the operating room. AI is becoming incredibly influential for surgical decision-making to help surgeons make better projections about the implications of surgical operations by considering different sources of data such as patient health conditions, disease natural history, patient values, and finance. Although the application of artificial intelligence in healthcare settings is rapidly increasing, its mainstream application in clinical practice remains limited. The use of machine learning algorithms in thoracic surgery is extensive, including different clinical stages. By leveraging techniques such as machine learning, computer vision, and robotics, AI may play a key role in diagnostic augmentation, operative management, pre-and post-surgical patient management, and upholding safety standards. AI, particularly in complex surgical procedures such as cardiothoracic surgery, may be a significant help to surgeons in executing more intricate surgeries with greater success, fewer complications, and ensuring patient safety, while also providing resources for robust research and better dissemination of knowledge. In this paper, we present an overview of AI applications in thoracic surgery and its related components, including contemporary projects and technology that use AI in cardiothoracic surgery and general care. We also discussed the future of AI and how high-tech operating rooms will use human-machine collaboration to improve performance and patient safety, as well as its future directions and limitations. It is vital for the surgeons to keep themselves acquainted with the latest technological advancement in AI order to grasp this technology and easily integrate it into clinical practice when it becomes accessible. This review is a great addition to literature, keeping practicing and aspiring surgeons up to date on the most recent advances in AI and cardiothoracic surgery.

## Highlights

• This literature review tells about the role of Artificial Intelligence in Cardiothoracic Surgery.
• Discussed the future of AI and how high-tech operating rooms will use human-machine collaboration to improve performance and patient safety, as well as its future directions and limitations.
• Vital for the surgeons to keep themselves acquainted with the latest technological advancement in AI order to grasp this technology and easily integrate it into clinical practice when it becomes accessible.

## 1 Artificial intelligence in surgery

Artificial intelligence (AI) can be defined as the study of algorithms that give an ability to reason to a machine and help it to perform cognitive tasks such as problem solving, decision-making, and word recognition [1]. With the blooming of the technological revolution, AI has become the centerpiece of both popular and academic literature. Today where data is key and technology is a major revolutionizing force, there is a shift in productivity like that of the industrial revolution [2]. Surgery stands to benefit from this technological growth as well, with a promising future for AI in surgery. There are four core subfields of AI in surgery namely machine-learning, natural language processing, artificial neural networks, and computer vision [1]. Machine-learning may seem very non-intuitive to the reader at first, as we are accustomed to human learning and machines being operators under human command. Machine learning (ML) basically recognizes patterns in enormous amount of data that may be imperceptible to the human mind, labels the data, and makes accurate predictions [3]. The role ML plays in surgery is evident from its ability to accurately predict surgical site infections and predict lung cancer staging, outperforming approaches based on clinical guidelines to unprecedented levels thought to be unattainable with conventional statistics [4,5]. Natural language processing (NLP) is a subfield of AI that emphasizes the computer's ability to not only understand human language but also infer meaning and sentiments from unstructured data [6]. In surgical patients, NLP has been able to detect phrases in operative reports that predicted anastomotic leaks after colorectal resection surgeries with sensitivities of 100% [7]. For example, ANN (sensitivity 89% and specificity 96%) have outperformed traditional risk prediction approaches like APACHE II (sensitivity 80% and specificity 85%) for pancreatitis severity 6 h after admission [8]. Computer vision describes the machine's ability to understand images and videos and has achieved capabilities comparable to human-level understanding in areas such as object and scene recognition [9]. For example, a real time analysis of a laparoscopic video of sleeve gastrectomy has noted missing or unexpected steps to 92.8% accuracy [10].

## 2 Role of AI in diagnostic augmentation

A sub-field of AI called ML can aid in diagnosis, augment surgical performance in the operating room (OR), and skill assessment. There are mainly two imaging-based applications of ML to thoracic surgery: automatic diagnosis of cardiovascular pathology and detection of patterns in imaging to detect thoracic pathologies like the classic chest X-ray example proposed by Wang et al. They used a chest x-ray database of 108,948 frontal view x-ray images from 32,717 unique patients and found out that it was able to distinctively diagnose 8 different pathologies [11]. Kusunose et al. investigated whether deep convolutional neural networks (DCNN) could detect regional wall motion abnormalities on echocardiography and achieved an area under the receiver operating characteristic (AUROC) of 0.99, outperforming physicians at the same task [12]. There are numerous applications of convolutional neural networks (CNN) to thoracic surgery, like detecting aortic diameter and volumetric analysis of the left ventricle to assess cardiac function. Image segmentation plays a pivotal role in assessing the anatomy of moving organs such as the heart and blood vessels by assigning a class label to image voxels so that they can be quantified. Bai W et al. [13] proposed an image sequence segmentation algorithm by combining a fully convolutional network with a recurrent neural network. Experiments of aortic magnetic resonance imaging studies demonstrated that the proposed method significantly improved accuracy and temporal smoothness, compared to a baseline method that only used spatial information. Another example where a video-based deep learning algorithm surpassed human expert performance is the EchoNet-Dynamic algorithm. Human assessment of cardiac function is associated with limited sampling of cardiac cycles and shows a lot of variability between observers but the EchoNet-Dynamic algorithm reported in a study by Ouyang et al. [14] surpassed human performance in assessing for cardiomyopathy and estimation of ejection fraction [14,15]. These studies show how useful AI can be for a thoracic surgeon's practice in translating to better patient care. Although the tasks of a thoracic surgeon in the OR are highly extensive and making an ML robot that can perform all the actions of a surgeon may be impossible, however, a study by Thananjeyan et al. shows that reinforcement learning (RL) powered robots can perform minor surgical tasks like simple suturing and highly precise surgical incisions [15]. Wijnberge et al. report in a recent randomized controlled trial that ML can detect the intra-operative incidence of hypotension in elective non-cardiac surgery, decreasing the median duration of hypotension from 32.7 min to just 8 min, that is a huge difference [16].

## 3 Human-machine teaming and computer vision in cardiothoracic surgery

Computer vision (CV) is a subdiscipline of AI engineering involved with the study of giving eyes to machines [17]. Computer vision is a promising AI method that can be used to monitor team dynamics in the OR [18]. Hao Xu et al. recently published a study focusing on the CV analysis of blood stains during thoracoscopic operations. They report that one of the major accidents that occur during thoracoscopic operations is massive bleeding accidents. These accidents lead to increased chances of mortality and prolonged hospital stays [19]. High throughput CV algorithms that could process many thoracic surgical videos were developed. They found a correlation between CV based-proportion of blood pixels (PBP) and bleeding volume by training the CV algorithm with thousands of pixels of either blood or non-blood that were selected randomly and labelled manually. PBP was only computed for some key reference frames but the CV algorithm could detect bleeding volume of whole surgery by just comparing frames from the surgery video with the reference frames of bleeding taught to the algorithm during its training. PBP can help guide postoperative fluid management, anticoagulation strategies, as high PBP values suggest a reduction in anticoagulation and vice versa, and selection of appropriate drainage tubes [19]. Cardiothoracic surgery requires top-notch technical (tying knots, suturing, and putting clamps) and non-technical skills like teamwork, communication, and awareness of the surrounding situation. In such conditions, Avrunin et al. [20] developed Smart checklists. These checklists can help the cardiothoracic surgical team, including surgical, anesthesiology, perfusion, and nursing staff. [20] Smart checklists reduce cognitive load and subsequent procedural errors by reminding the team about the next step in the procedure and by alerting the other team members not to disturb or increase cognitive load on specific members of the team during some high cognitive load scenarios [20]. Communication, coordination, and adaptation are qualities that are critical to human-machine teaming, but machines have not yet fully realized the unique human cognition for effective teaming. The rapid technological advancements in the recent era, especially in the field of AI, have offered new capabilities to machines to maximize their competencies in this regard [21]. According to Seeber et al. [22] rapid technological advancements have enabled machines to acquire transportable teamwork competencies that are critical to teams. For example, machines may leverage a theory of mind reasoning to build a computerized model of their teammates. Using this model, the machine can know what information is available to teammates and what would be the subsequent action of their human teammates, creating better coordination with the human counterparts in a team. This model will also be able to determine when and how best to communicate with teammates, further enhancing ability and trust in machines [[21], [22], [23], [24]]. Current AI research focuses on specific objectives to be achieved by machines and doesn't incorporate many findings from teaming and human-computer interface literature. For Example, Open AI Five is a five neural network model developed to beat top five human champions in a game, although neural network models performed best when not paired with other machines, they performed worst when partnered with humans. Examining such phenomenon Carroll M et al. found out that the main limiting factor for such behavior of the neural networks was initial machine training in conjunction with other machines [24].

## 4 Role of AI in preoperative performance and safety in cardiothoracic surgery

Chang Junior J et al. studied the role of artificial intelligence and reported that the sheer number of cardiac surgical interventions available for congenital cardiac diseases and the low volume of patients compared with adults make it hard to collect large amounts of data regarding pre-operative safety and efficacy for a single procedure [25]. The random forest model of artificial intelligence can learn from large pools of data and accurately predict individual death risks in patients with congenital heart disease. The findings of this model can assist patients, surgeons, and family members of patients in understanding the risks associated with a cardiac surgical intervention [25]. With the advent and integration of AI into clinical care, the traditional systems are replaced with more efficient and more accurate systems. Using deep and machine learning, AI helps in the pre-operative automation of clerical processes, provides assistance in how patients can be triaged and gives risk predictions during the COVID-19 pandemic where resources were already scarce, and cardiac surgery patients were facing delays in delivery of care [26]. Using machine learning techniques, Yoon et al. devised a new personalized method for prediction of risk both pre- and post-cardiac transplantation. The method is used to classify the heterogeneous cohort of patients and their interactions with each other across different zones of time. The method is so robust that it outperforms the best clinical scores and machine learning methods currently available. The same method can be applied to other fields of medicine and surgery [27]. Pre-operatively, Kwon et al. used the deep learning approach in AI for predicting the mortality of acute heart failure by an algorithm known as deep-learning-based artificial intelligence algorithm for predicting mortality of patients with acute heart failure (DAHF). The deep learning algorithm was so efficient and reliably accurate that it could predict the one-year and three-year mortality of acute heart failure patients more accurately than the existing scores and machine learning methods [28]. A study by Kilic et al. revealed that there is an increase in the use of durable left ventricular assist devices (LVAD) being implanted in the United States but there was no widely used risk stratification tool for LVAD therapy. By utilizing the Interagency Registry for Mechanically Assisted Circulatory Support (INTERMACS) database they studied the 90-day and 1-year mortality rates following primary LVAD implantation using both logistic regression and machine learning approaches and found that machine learning models were both well-calibrated and had improved discriminatory capability as compared to logistic regression [29]. Wang et al. developed and validated a machine learning method that can predict the amount of red blood cell transfusions required for a cardiothoracic surgery and showed excellent results [30]. Artificial intelligence and virtual reality can give a 3- dimensional view of whole pulmonary anatomy with its intricate parts to the cardiothoracic surgeon to preoperatively get a better insight into the individual patient's anatomy [31].

## 5 Role of artificial intelligence in intra operative performance and safety in cardiac/cardiothoracic surgery

Humans have made impeccable technological advancements in the past decades, and these advancements have also had an impact on the contemporary OR with artificial intelligence being at the forefront of these technological achievements. Artificial intelligence benefits the surgical team as an augmentation to the normal human cognition in the OR by processing complex computations to provide a meaningful result that helps the surgical team and the patient. Artificial intelligence is used to monitor and assess team as well as individual performance throughout the surgery which can help guide better patient outcomes by addressing the underlying areas of improvement specified by the artificial intelligence system [32]. Goldenberg et al. showed that traditionally, operative quality was assessed by retrospectively reviewing patient medical records, which were inundated with biases like recall bias and non-compliance on the part of the patient. Artificial intelligence allows for a highly sensitive prospective intraoperative approach called the OR black box system, which focuses from major points to the minutiae by collecting and integrating data regarding events that may cause patient harm at a magnitude not feasibly possible by a human [33]. Azari et al. used computer vision to assess individual task performance during an ongoing surgical operation. The computer vision analysis assessed surgeon techniques more reliably and objectively than the individual assessments made by surgical experts [34].Artificial intelligence can accurately predict the risk of hypoxemia during cardiac surgery. Lundberg SM et al. revealed that machine learning can predict the future risk of intraoperative hypoxemia and provide an explanation of the risk factors to an accurate level consistent with literature. Artificial intelligence can amplify the current anesthesiologist's prediction of hypoxemia during surgery by 15%–30%; an almost double when predicting events of hypoxemia [35]. Ali et al. shows in their study that surgeons often either underestimate or overestimate the time required for a surgery, leading to either underutilization of the financial and human resources in the operating room or over-burdening of the resources when time required for a surgery is over-estimated. Artificial intelligence can bridge this gap by accurately predicting surgery duration leading to positive outcomes in the form of proper and commensurate resource utilization [36]. Even now, there is an absence of real-time intraoperative imaging techniques for the right ventricle during cardiac surgery. Muzio L et al. used an AI-based video kinematic evaluation technique for the right ventricle during surgeries for tetralogy of Fallot cases, using a supervised machine learning model to predict the outcome of the intervention and its success upon chest closure [37].

## 6 Role of AI in post-operative management

Using personalized risk factor assessment is a future trend in precision medicine and using the help of AI, especially supervised machine learning can give us a feasible solution to it. Individualized risk assessment by AI models can help us if the results are accurate, predictive, and consistently reproducible each time the assessment is performed. Chang et al. used the Naïve Bayes (NB) - algorithm assisted prediction system to assess the need of patients for high concentration oxygenation, ICU care and the risk of inability to wean-off the ventilator immediately following lung resection surgery with remarkable results of 100% of the patients agreeing that digitalization improved their understanding of the needs and the risks predicted by the NB-algorithm assisted prediction system [38]. Tseng et al. developed and validated machine learning models for 94 pre- and intraoperative features to predict cardiac surgery-associated acute kidney injury (CSA-AKI), which occurs in approximately 22% of cardiac surgery cases. Previous studies and risk scores used categorical division of parameters pertaining to the risk which failed to preserve variability in continuous data. The machine learning method to determine the risk of CSA-AKI was successful and the study also demonstrated that intraoperative time-series and other features are important for acute kidney injury prediction [39]. Fernandes et al. used machine learning methods and also added cardiopulmonary bypass-specific intraoperative hypotension (CBP-specific IOH) as a parameter to the preoperative score leading to results that machine learning methods that incorporated the CBP-specific IOH to demonstrate great predictive ability of post-surgical mortality [40]. Mufti et al. studied different machine learning methods to develop models that could predict the occurrence of delirium after cardiac surgery and found that machine learning methods can help in revealing the hidden patterns in delirium causation and better predicting its occurrence [41].

## 7 Limitations, ethical issues and needs in future

Although AI has shown highly encouraging results in all fields of patientcare, many issues need to be addressed before it can be used in the daily routine of a cardiothoracic surgeon. With the advent of AI, the privacy of patients' data is a big concern [42]. Price et al. argue that overprotection with privacy can lead to a halting or braking in technological innovation, while on the other hand, allowing privacy to drive secrecy can lead to less trust in the technological achievements brought about by it [43]. He et al. argue that transparent and accurate input is required for the AI systems to generate accurate results. Another reason why transparency is related to functioning of the AI system is that if patient care providers can accurately work out how a machine came to a conclusion, this is a way in which it can be tested whether the reasoning is sound. Opaque AI systems do work based on algorithms, but there is no way a patient care provider can know how a system came to such a conclusion. This makes it harder to test for the soundness of its reasoning and integration of a particular a technology into the patient care [44]. Perhaps the biggest shortcoming of ML lies in its lack of interpretability of the produced outcomes. Normal regression techniques can be tested and relationships can be seen such that increasing or decreasing input feature x affects the output feature y in different ways, but AI uncovers non-linear and highly complex associations in and between datasets at a scale so humongous, that it’s hard for the human brain to compute. Hence the way AI finds these associations is also a mystery to care providers [45].

## 8 Conclusion

Recent technological advances and diligent research have resulted in intelligent technology by the development of innovative computer algorithms and the augmentation of the application of human cognitive models for AI. AI technologies are becoming increasingly useful and are being integrated into multidisciplinary health-care models, such as perioperative medicine, smart operating theatres, robotic interventions, intraoperative management, maintaining patient safety, identifying risk factors, and postoperative rehabilitation. There is promising future of AI that can open a new world for cardiothoracic surgery, however there are certain barriers to mass-implementation of these novel technologies. The transparency of patients' data, establishment of secure algorithms, human trials, and ethical concerns regarding involvement of extensive machinery in complex surgeries can limit the growth of this remarkable technology. It is critical for practicing surgeons and aspiring surgeons to stay current on technology advancements in order to better comprehend them and integrate them into clinical practice for improved outcomes and advancement of specialty.

## Conflicts of interest

Nil.

## Sources of funding

Nil.

## Ethical approval

Ethical approval was not required as it is a literature review.

## Consent

The informed consent from the patients was obtained considering Helsinki's Declaration.

## Author contribution

Contributorship Statement:

1. The main concept was determined by Hassan Mumtaz
2. Collection of data is done by Hassan Mumtaz and Muhammad Saqib
3. Data is analyzed and interpreted by Muhammad Saqib, Pakiza Muskan
4. Writing of the manuscript is done by Farrukh Ansar and Durafshan Zargar
5. Manuscript editing is done by Madiha Hameed and MOHAMMAD HASAN

## Registration of research studies

1. Name of the registry: Research Registry
2. Unique Identifying number or registration ID: reviewregistry1380
3. Hyperlink to your specific registration (must be publicly accessible and will be checked: https://www.researchregistry.com/browse-the-registry#registryofsystematicreviewsmeta-analyses/registryofsystematicreviewsmeta-analysesdetails/62b05c61f43315001e03a322/

## Guarantor

Hassan Mumtaz.

## Funding

Nill.

## Declaration of competing interest

Nill.
